# Genome-wide investigation of the *ZF-HD* gene family in Tartary buckwheat (*Fagopyrum tataricum*)

**DOI:** 10.1186/s12870-019-1834-7

**Published:** 2019-06-11

**Authors:** Moyang Liu, Xiaoxiang Wang, Wenjun Sun, Zhaotang Ma, Tianrun Zheng, Li Huang, Qi Wu, Zizhong Tang, Tongliang Bu, Chenglei Li, Hui Chen

**Affiliations:** 1 College of Life Science, Sichuan Agricultural University, Ya’an, China; 2 School of Agriculture and Biolog, Shanghai Jiao Tong University, Shanghai, China

**Keywords:** Tartary buckwheat, ZF-HDs, Genome-wide, Fruit development, Expression patterns

## Abstract

**Background:**

ZF-HD is a family of genes that play an important role in plant growth, development, some studies have found that after overexpression *AtZHD1* in *Arabidopsis thaliana*, florescence advance, the seeds get bigger and the life span of seeds is prolonged, moreover, *ZF-HD* genes are also participate in responding to adversity stress. The whole genome of the *ZF-HD* gene family has been studied in several model plants, such as *Arabidopsis thaliana* and rice. However, there has been little research on the *ZF-HD* genes in Tartary buckwheat (*Fagopyrum tataricum*), which is an important edible and medicinal crop. The recently published whole genome sequence of Tartary buckwheat allows us to study the tissue and expression profiles of the *ZF-HD* gene family in Tartary buckwheat on a genome-wide basis.

**Results:**

In this study, the whole genome and expression profile of the *ZF-HD* gene family were analyzed for the first time in Tartary buckwheat. We identified 20 *FtZF-HD* genes and divided them into MIF and ZHD subfamilies according to phylogeny. The ZHD genes were divided into 5 subfamilies. Twenty *FtZF-HD* genes were distributed on 7 chromosomes, and almost all the genes had no introns. We detected seven pairs of chromosomes with fragment repeats, but no tandem repeats were detected. In different tissues and at different fruit development stages, the *FtZF-HD* genes obtained by a real-time quantitative PCR analysis showed obvious expression patterns.

**Conclusions:**

In this study, 20 *FtZF-HD* genes were identified in Tartary buckwheat, and the structures, evolution and expression patterns of the proteins were studied. Our findings provide a valuable basis for further analysis of the biological function of the *ZF-HD* gene family. Our study also laid a foundation for the improvement of Tartary buckwheat crops.

**Electronic supplementary material:**

The online version of this article (10.1186/s12870-019-1834-7) contains supplementary material, which is available to authorized users.

## Background

Specialized genetic networks regulate plant growth by encoding various proteins. Proteins containing transcription factors that bind to specific nucleotide sequences play an important role in different stages of plant growth, flowering, fruiting, and resistance to stress [1, 2]. A homeodomain (HD), as an NDA domain (BD), has 60 conserved amino acid sequences and encodes a homeobox (HB) gene in all eukaryotic transcription factors [3]. The 60 amino acids of the homeodomain fold into a characteristic three-helix structure called recognition helix, which attaches to the main sulcus of DNA to form a special connection to DNA [4, 5]. The HD protein is involved in the development of plants and animals by regulating the expression pattern of target genes [3]. Most HD proteins are related to the protein-protein interactions and other domains/motifs with regulatory functions [6]. Proteins with homologous domains are divided into six different families according to different themes: leucine zipper-associated HD (HD-Zip), zinc finger motif-associated HD (ZF-HD), WUSCHEL-related homeobox (WOX), Bell-type HD, finger-like domain associated with an HD (PHD finger), and knotted-related homeobox (KNOX) proteins [7].

The zinc finger structure is an important structure and is composed of zinc ions and cysteine or histidine (in most cases) [8]. The zinc finger, as an important motif, is widely found in a variety of regulatory proteins, can specifically bind to DNA/RNA sequences, and participates in protein interactions [9, 10]. Zinc finger units are divided into many categories according to Cys and differences in residues, such as C3hC2H2 and C2C2 [8]. As a family, the zinc finger homeodomain (ZF-HD) proteins, containing HD proteins and a zinc finger related to the homeodomain, were first identified in the C4 plant *Flaveria* [11].

At present, the *ZF-HD* gene family has been identified in *Arabidopsis thaliana*, rice (*Oryza sativa*) and tomato (*Solanum lycopersicum*) [12–14]. *Arabidopsis thaliana* has 17 ZF-HD members, and they act as transcription factors, have unique physiological characteristics, and play a very important role in the development of flowers [11]. Drought, salinity and abscisic acid (ABA) can induce AtZHD1 to bind to the ERD1 promoter region [15] Three genes were found in the *ZF-HD* gene family of *Arabidopsis thaliana*, and its mini zinc finger (*MIF*) gene and protein sequence encoded by congeners are highly similar to the ZF domain of the ZF-HD protein but have no HD domain [12, 16]. The phylogeny and sequence analyses of *MIF* and *ZHD* genes were conducted, suggesting that the *ZHD* gene has plant specificity and that almost all genes have no introns [11]. Until now, the origin and evolution of the *ZHD* and *MIF* gene populations remain unclear [12]. In soybean, *GmZF-HD1* and *GmZF-HD2* bind to the promoter region of the gene encoding calmodulin subtype 4 (*GmCaM4*), and the expression of *GmZF-HD1* and *GmZF-HD2* increased after inoculation with pathogenic bacteria [17]. In tomato, the *ZF-HD* gene family was found to be related to fruit development as well as to stress [14]. The *ZF-HD* gene family may play a similar role in Tartary buckwheat.

Common buckwheat (*Fagopyrum esculentum*) is produced in southwestern China and has spread to all continents. Tartary buckwheat (*Fagopyrum tataricum*) grows in the mountainous areas of southwestern China, northern India, Bhutan and Nepal [18]. Tartary buckwheat is currently the only widely grown single-sex only food crop, has a balanced essential amino acid composition in its seed protein, and has a total protein content that is higher than that in many food crops [8, 19]. The *ZF-HD* gene family has been widely studied in many plants, some studies have found that after overexpression *AtZHD1* in *Arabidopsis thaliana*, florescence advance, the seeds get bigger and the life span of seeds is prolonged [12, 20], moreover, *ZF-HD* genes are also participate in responding to adversity stress [21]. However, the *ZF-HD* gene family in Tartary buckwheat has not been studied, at present, the *ARF, AP2, NAC, MADS* genes family have been studied deeply in Tartary Buckwheat [22–25]. Because of the important role of the *ZF-HD* gene in various physiological processes, it is of great significance to systematically study the Tartary buckwheat *ZF-HD* family. The recently completed genome sequence of Tartary buckwheat provides an opportunity to reveal the tissue, expression and evolution characteristics of the *ZF-HD* gene family in Tartary buckwheat at the whole genome level. In this paper, the exon-intron structure, motif composition, genomic structure, chromosome location, sequence homology and expression pattern of 20 Tartary buckwheat *ZF-HD* genes are introduced in detail. In addition, the phylogenetic relationship between the *ZF-HD* gene family in *Arabidopsis thaliana* and Tartary buckwheat was compared. Through global expression analysis, the degree of participation of *ZF-HD* gene family members in different biological processes of Tartary buckwheat was determined. The role of the *FtZF-HD* gene in the development of buckwheat fruit was studied in detail, which provided a valuable clue for the functional characterization of the buckwheat *ZF-HD* gene family members in the growth and development of Tartary buckwheat.

## Results

### Identification of the *FtZF-HD* genes in Tartary buckwheat

To identify the *FtZF-HD* genes in Tartary buckwheat, all possible *FtZF-HD* members in the Tartary buckwheat genome were mined using two BLAST methods, multiple *FtZF-HD* genes from the Tartary buckwheat genome were isolated by these two methods, and since the buckwheat genome was sequenced using a genome-wide shotgun strategy, some of these *FtZF-HD* genes may be redundant even though they were on different scaffolds. In this study, we identified a total of 20 *ZF-HD* genes, and we named them *FtZHD1*~*FtZHD17* and *FtMIF1~FtMIF3* based on their physical location on the chromosomes (Additional file 2: Table S1).

For the Tartary buckwheat *FtZF-HD* proteins, FtZHD14 was the smallest protein with 83aa, and the largest protein was *FtZF-HD*5 (330aa) (Additional file 2: Table S1). The molecular weight of the proteins ranged from 9.33 kda to 34.95 kda; PI from 4.94 (*FtMIF1*) to 10.15 (*FtZHD10*). The predicted subcellular localization results showed that all proteins are located in the nuclear region.

### Phylogenetic analysis of the ZF-HD gene family in Tartary buckwheat

**Fig. 1:**
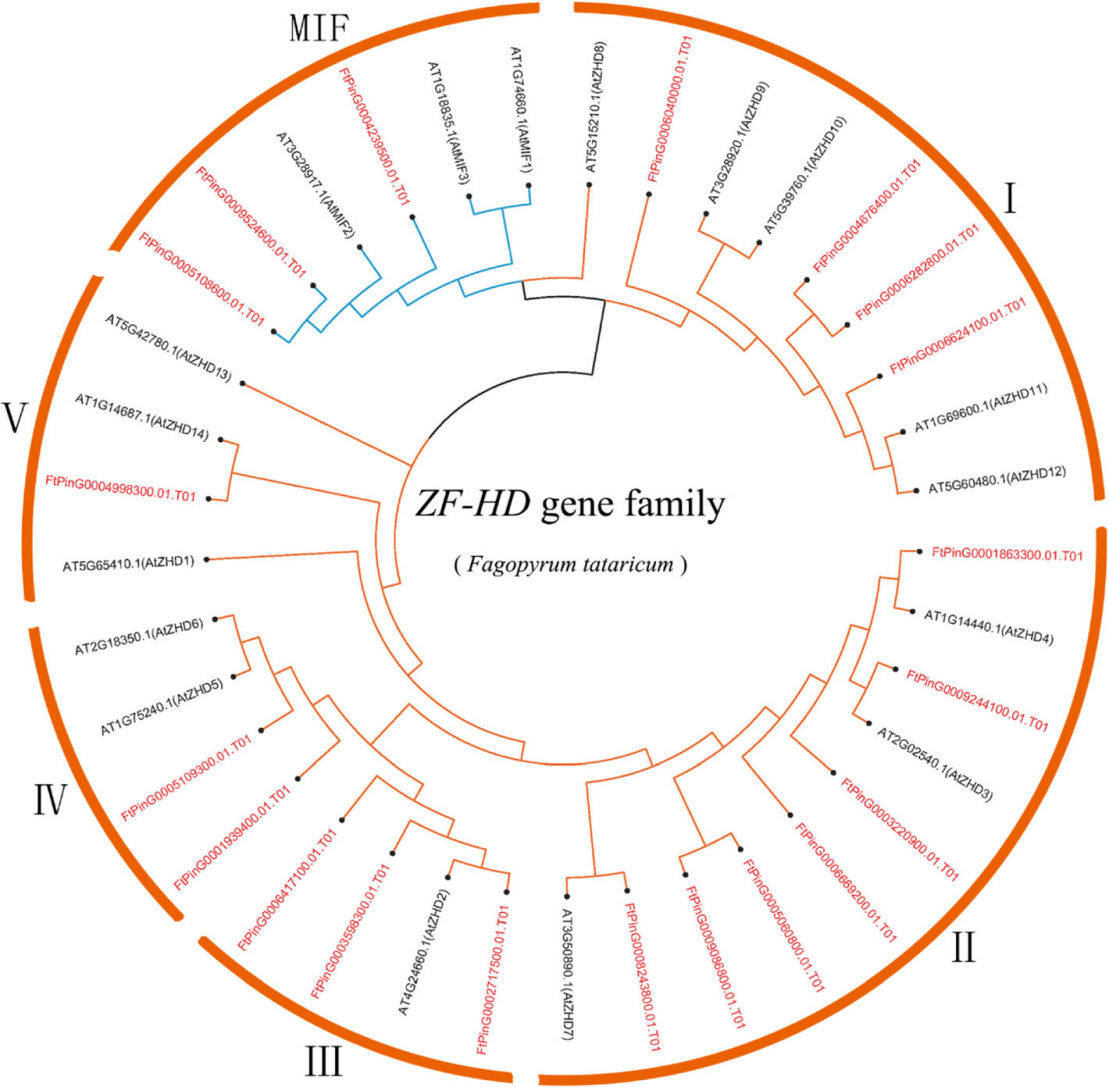
Unrooted phylogenetic tree based on the relationship between Tartary buckwheat and Arabidopsis. Each arc represents a different group (or subgroup) of *ZF-HD*

To investigate the phylogenetic relationship of the ZF-HD proteins in Tartary buckwheat, the neighbor-joining (NJ) method of Geneious R11 was used to construct a phylogenetic tree consisting of *Arabidopsis thaliana* (17 gene) and Tartary buckwheat (20 gene). The phylogenetic distribution indicated that the *FtZF-HD* gene family can be divided into two subfamilies: *MIF* and *ZHD. ZHD* was further divided into five parts *(ZHDI, ZHDII, ZHDIII, ZHDIV* and *ZHDV)* (Fig.1). The number of *ZHD IV* genes was the least (5%) and that in the *ZHD II* gene family was the highest (35%). In addition to *FtZHD17* and *FtZHD12*, the *ZF-HD* gene family of Tartary buckwheat was similar to the *ZF-HD* family of *Arabidopsis thaliana*; the highest degree of similarity was 78.85% (*FtZHD14*), and the lowest was 38.06% (*FtZHD9*). *FtZHD17* and *FtZHD12* were more similar to rice *OsZHD1*, 44.71 and 49.27%, respectively (Additional file 2: Table S1). Moreover, the results showed that the *ZF-HD* gene was amplified in Tartary buckwheat.

### Structural analysis and motif composition of the Tartary buckwheat ZF-HD gene family

**Fig. 2:**
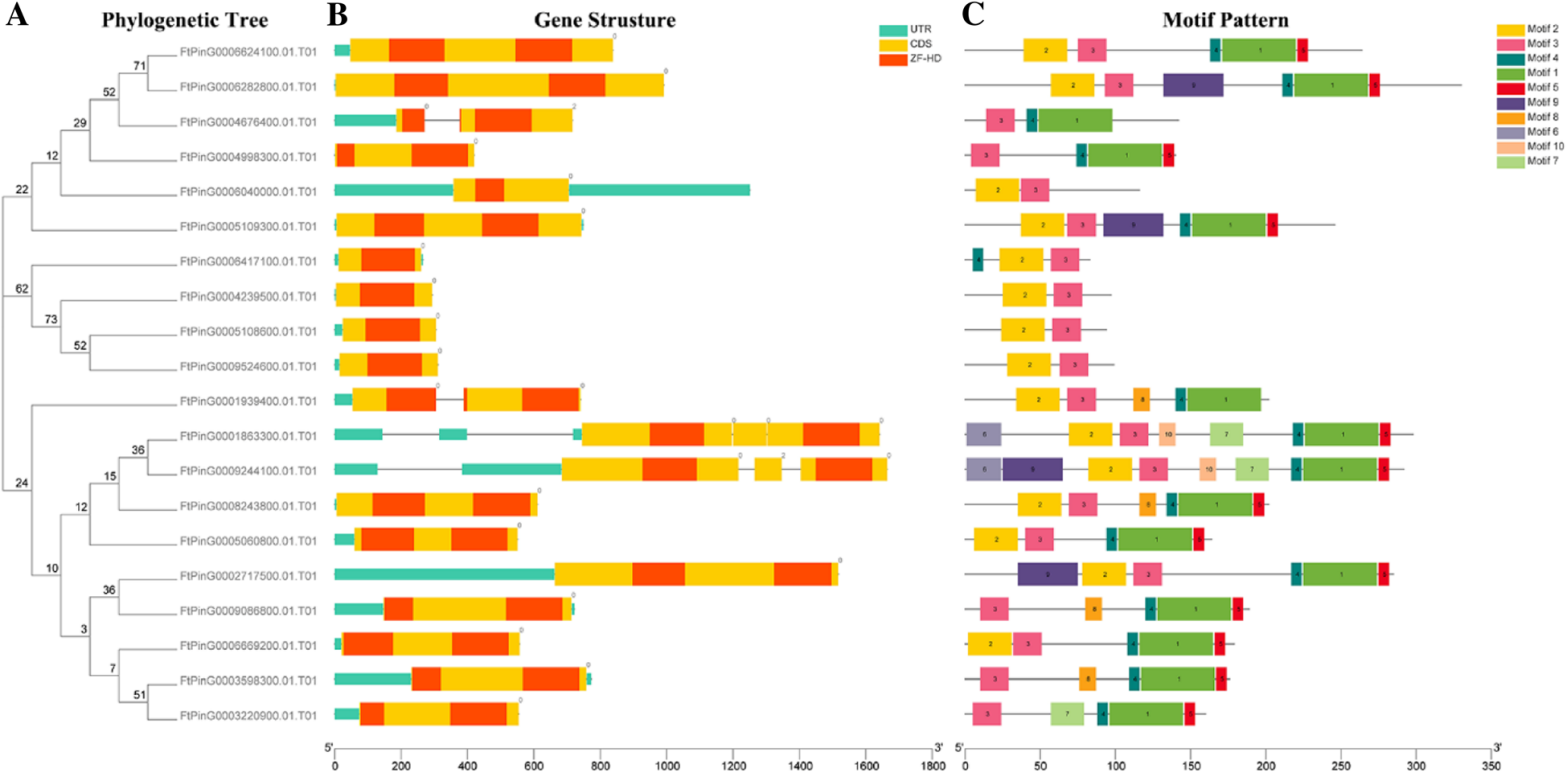
Phylogenetic relationships, gene structures and architectures of the conserved protein motifs in *ZF-HD* genes from Tartary buckwheat. (**a**) The phylogenetic tree was constructed based on the full-length sequences of Tartary buckwheat ZF-HD proteins using Geneious R11 software. (**b**) Exon-intron structures of the Tartary buckwheat *ZF-HD* genes. Green boxes indicate untranslated 5′- and 3′-regions; yellow boxes indicate exons; and black lines indicate introns. The ZF-HD domains are highlighted by red boxes. The number indicates the phases of the corresponding introns. (**c**) The motif composition of the Tartary buckwheat ZF-HD proteins. The motifs, numbered 1–10, are displayed in different colored boxes. The sequence information for each motif is provided in Additional file 3: Table S2. The length of the protein can be estimated using the scale at the bottom

To further understand the structural components of the *FtZF-HD* gene, we compared the corresponding genomic DNA sequences, and we obtained the exon and intron structures of the *FtZF-HD* genes (Fig. 2b). Interestingly, most of the *FtZF-HD* genes (16 out of 20) did not have introns, but two of the other four genes did have an intron. The remaining two genes had two introns each. Moreover, the location of the intron of the two genes was very close, and there may be a replication relationship between the two introns. Most genes with similar evolutionary relationships have similar exon-intron structures. When genes do not have introns, it means that they are not easily connected and have relatively conserved functions. This result is consistent with the result from the previously reported case in which the *ZF-HD* gene had almost no introns [11]. The intron of the Tartary buckwheat *ZF-HD* gene is inherited via evolution or obtained by mutations during evolution, and whether the appearance of introns has an effect on the original function of these genes is a question that needs to be further studied.

To further study the characteristic region of the *FtZF-HD* protein, the structures of 20 *FtZF-HD* proteins were analyzed by online MEME analysis. According to the results of the MEME analysis, a schematic map was constructed to characterize the structure of the *FtZF-HD* protein. We identified 10 conserved motifs, named motifs 1 to 10 (Fig. 2c). It is worth noting that all *FtZF-HD* genes had motif3 domains, and most (60%) had motif2 domains. Interestingly, motif4 was a domain that was specific to the ZHD family, and motif1 and motif5 were detected in almost all *ZHD* genes. Motif6 and motif10 were detected specifically in the *FtPinG0001863300.01* and *FtPinG0009244100.01*genes. Motif8 and motif9 existed in four genes each. Motif7, motif8, and motif9 only existed in a few genes. Compared with the *MIF* gene family, the *ZHD* gene family showed obvious differences, and the functional differences in the *ZHD* gene in Tartary buckwheat were probably due to the subfamily-specific distribution of conserved motifs. The same motifs in three *MIF* genes and the same motifs in several subpopulations of *ZHD* indicated that there were conserved motifs in the subpopulations, but the function of these conserved motifs remains to be clarified.

### Chromosome distribution and synchronous analysis of the *FtZF-HD* genes

**Fig. 3:**
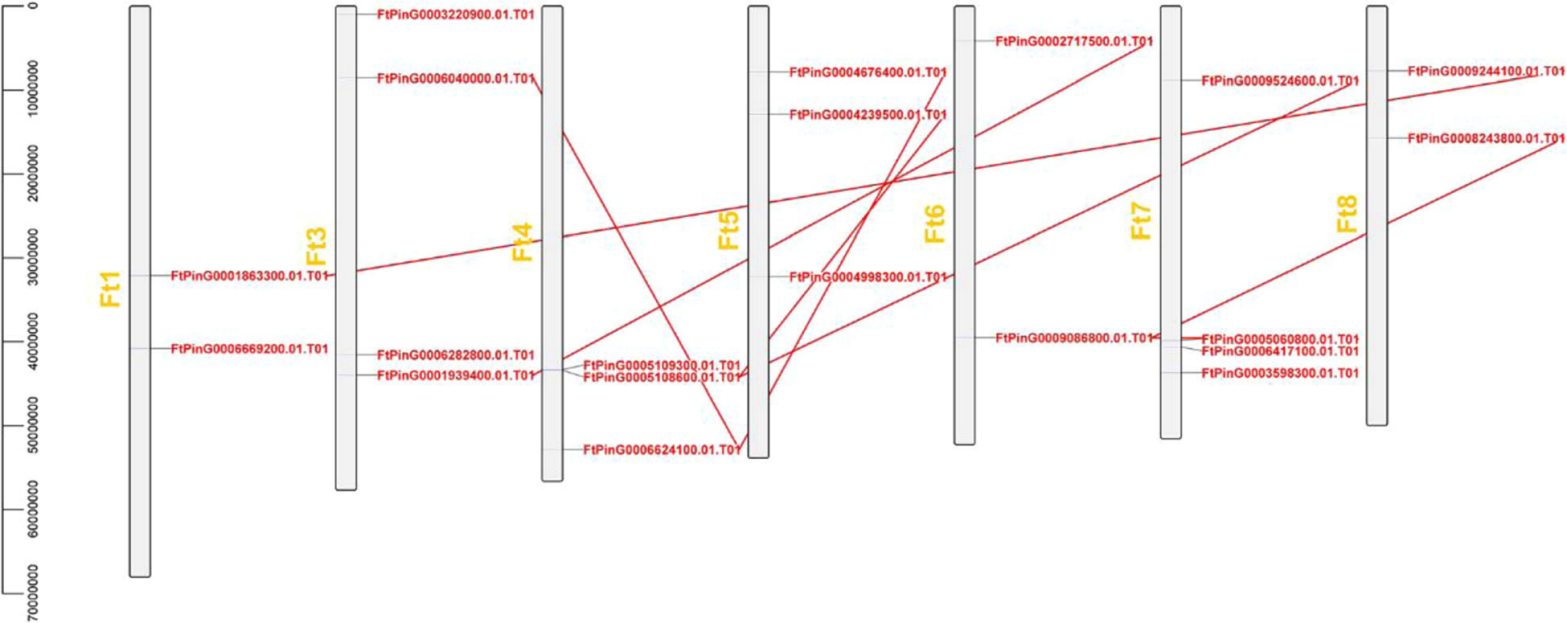
Schematic representations of the chromosomal distribution and inter-chromosomal relationships of the Tartary buckwheat *ZF-HD* genes. The red color denotes the *ZF-HD* gene, and the red line indicates that there is a fragment repetition of the two genes. The chromosome number is marked in yellow

There was an uneven distribution of the *FtZF-HD* genes on 7 buckwheat chromosomes (except Ft2). Chromosome 3 and chromosome 7, which were the two chromosomes with the most *ZF-HD* genes, each contained four *ZF-HD* genes, and three chromosomes had the lowest number of *ZF-HD* genes (2 *ZF-HD* genes)(Fig. 3). Gene replication plays an important role in the occurrence of new functions and gene amplification. To determine the fragment replication events between the genes, we adopted the standard [26]. When the query coverage and consistency of the candidate genes are ≥80, they are thought to be repetitive genes. Chromosomal regions within the 200 kb range of two or more genes are defined as tandem replication events. Therefore, the analysis of Tartary buckwheat gene duplication showed that no tandem repeat gene was found on the chromosomes of Tartary buckwheat; however, 12 genes were involved in fragment repeat events, and the *ZF-HD* gene on chromosome 4 was involved in the most fragment repeat events (Fig. 3). These results suggest that some *FtZF-HD* genes may be produced by repeated fragments of the gene, and these replication events are the main driving force in the evolution of *FtZF-HD*.

**Fig. 4:**
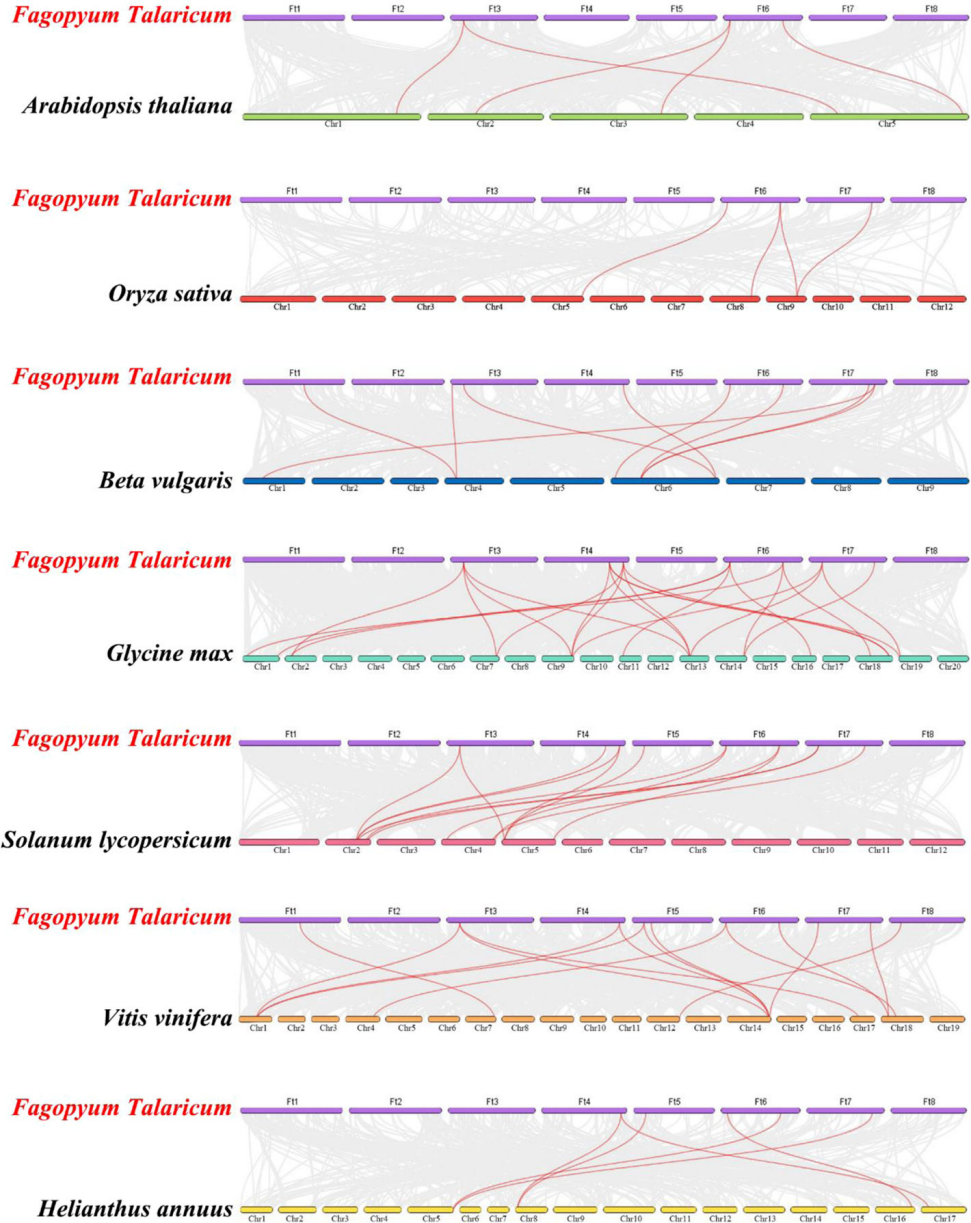
Synteny analysis of the *ZF-HD* genes between Tartary buckwheat and seven representative plant species. Gray lines in the background indicate the collinear blocks within the Tartary buckwheat and other plant genomes, while the red lines highlight the syntenic *ZF-HD* gene pairs

To further infer the phylogenetic relationship between Tartary buckwheat and dicotyledonous plants, we analyzed the collinear relationships between seven plants and Tartary buckwheat (Fig. 4). The results showed that 23 *FtZF-HD* genes were collinear with soybean genes, followed by grape (15), tomato (13), beet (9), sunflower (6), Arabidopsis (5), and rice (4). Some *FtZF-HD* genes were associated with at least two pairs of homologous genes, especially the ZF-HD genes of Tartary buckwheat and soybean, such as FtPinG0006040000.01, FtPinG0006624100.01and FtPinG0002717500.01, which may play an important role in the evolution of the ZF-HD gene family (Additional file 4: Table S3). The relationships between Tartary buckwheat and soybean, tomato, sugar beet and grape were similar. The comparison of the ZF-HD gene analysis between Tartary buckwheat and other plants is of great significance for establishing the genetic relationship among species and predicting the function of genes.

### Evolution analysis of the *FtZF-HD* gene family within several different species

**Fig. 5:**
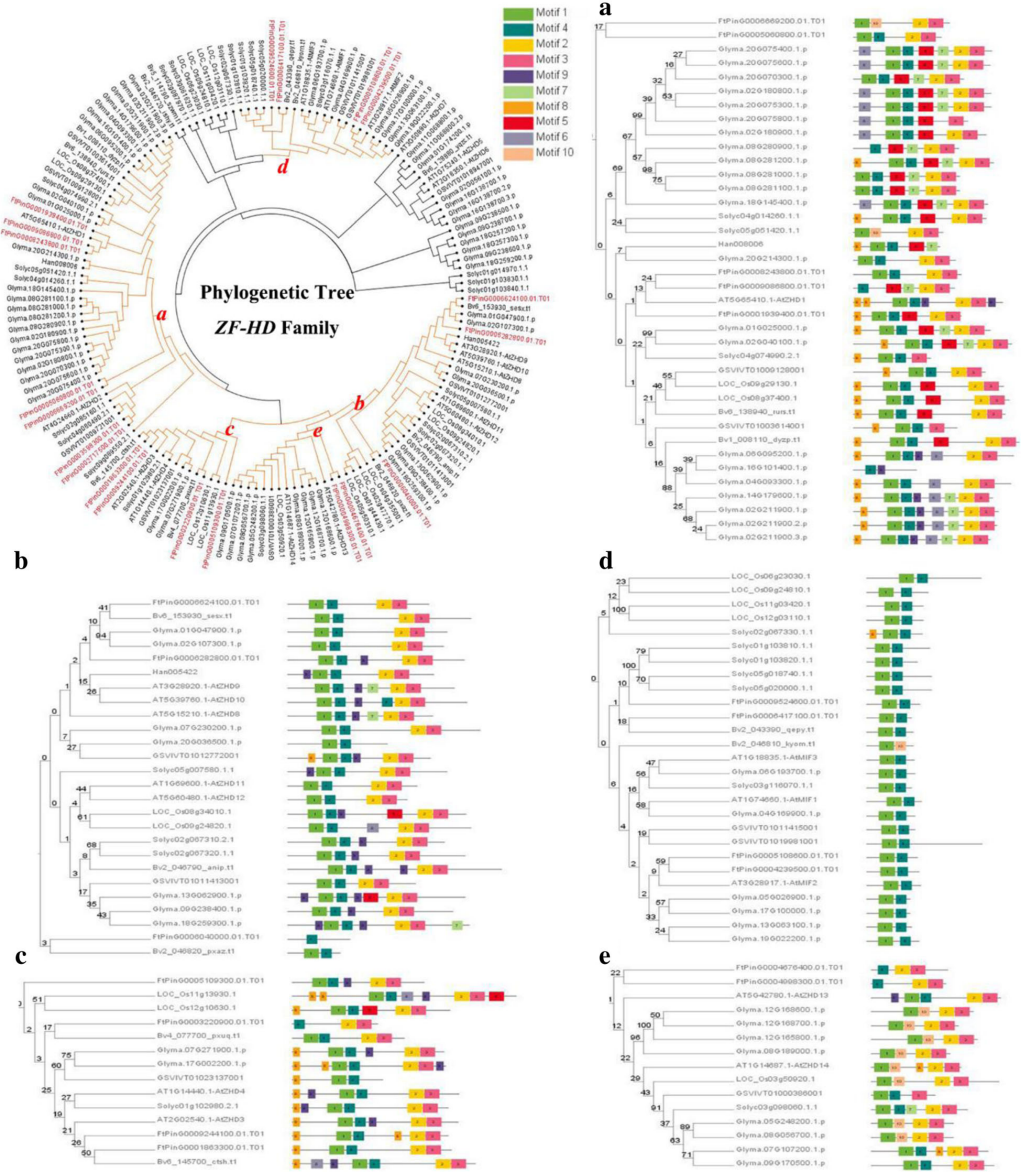
Phylogenetic relationships and motif compositions of the *ZF-HD* proteins from four different plant species. Left panel: an unrooted phylogenetic tree constructed using Geneious R11 with the neighbor-joining method. Right panel: distribution of the conserved motifs in the ZF-HD proteins. The different colored boxes represent different motifs and their positions in each ZF-HD protein sequence

The number of *FtZF-HD* genes identified was similar to that of *Arabidopsis thaliana* (17), rice (15, 14] and other plant *ZF-HD* gene families. But the genome size of the three species was very different (*Tartary buckwheat*, 516 Mb; *Arabidopsis thaliana, 125* Mb*; rice, 466* Mb). This result indicated that the *ZF-HD* gene family remained stable in different species during long-term evolution. Based on the existing ZF-HD genes of Tartary buckwheat, the replication and diversity of the *ZF-HD* genes during evolution were further studied. We constructed a phylogenetic tree with the *ZF-HD* protein sequences of Tartary buckwheat, a monocotyledonous plant (rice) and six dicotyledonous plants (*Arabidopsis thaliana*, soybean, tomato, beet, grape and sunflower) using the neighbor-joining method of Geneious R11. The ZF-HD proteins were divided into five groups according to the phylogenetic tree (Fig. 5). Group A contained the most *ZF-HD* genes (7 genes) in Tartary buckwheat, Group C and Group D contained 4 each, and Group Band Group E contained the least (2 genes).

We also use MEME web servers to search for conserved motifs shared by the ZF-HD proteins. The results showed (Fig. 5) that 10 different conserved motifs were identified. The genes of the same group had similar motifs, such as those in group d, which suggests potential functional similarities among the ZF-HD proteins. Motif 1 and motif 4 were conserved motifs shared by almost all ZF-HD proteins, indicating that *ZF-HD* had some highly conserved domains. Motif2 and motif3 were conserved domains shared by almost all ZF-HD proteins except for group d; therefore, group d may have evolved from other genes after the loss of motif2 and motif3 or other genes evolved after acquiring motif2 and motif3.

### Expression patterns of the *FtZF-HD* genes in different plant tissues

**Fig. 6:**
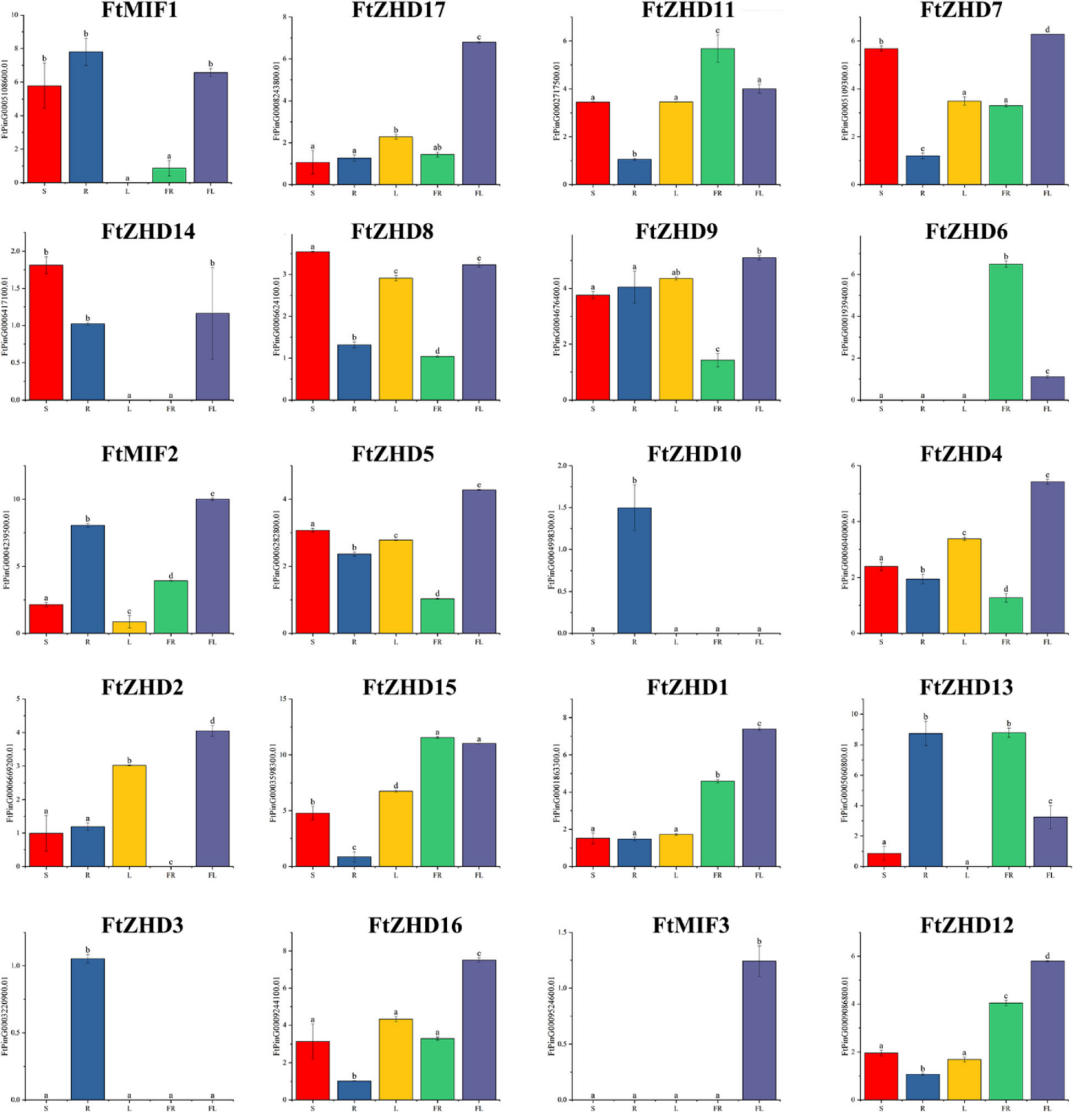
Tissue-specific gene expression of 20 Tartary buckwheat *ZF-HD* genes. The expression patterns of 20 Tartary buckwheat *ZF-HD* genes in the flower, leaf, root, stem and fruit tissues were examined by a qPCR assay. Error bars were obtained from three measurements. Lowercase letter(s) above the bars indicate significant differences (α = 0.05, LSD) among the treatments

To study the physiological function of the *FtZF-HD* genes, a real-time PCR technique was used to detect the time of expression of individual members of the gene family. The accumulation of *FtZF-HD* transcription products in the roots, stems, leaves, flowers, fruits and other tissues of Tartary buckwheat was evaluated (Fig. 6). The results showed that the transcriptional abundance of the *FtZF-HD* gene varied greatly in the different tissues and organs, suggesting that the *FtZF-HD* gene family has many functions in the growth and development of Tartary buckwheat. Some *FtZF-HD* genes that were expressed in Tartary buckwheat stem had tissue specificity, gene *FtMIF3* was expressed only in flowers, *FtZHD10* and *FtZHD3* were expressed only in the roots, *FtZHD2* was only not expressed in the fruit, and *FtZHD13* and *FtMIF1* were not expressed in leaves. Four *FtZF-HD* genes (*FtZHD11/ FtZHD6/ FtZHD15/ FtZHD13*) in Tartary buckwheat fruit had high levels of expression. The expression of 11 *FtZF-HD* genes (*FtZHD1/2/4/5/7/9/12/16/17* and *FtMIF2/3*) in the flowers was higher than that in other organs. This study found that only five *FtZF-HD* genes were expressed more in the reproductive organs than in the other tissues (*FtZHD*1/6/11/12/15) (Fig. 6).

**Fig. 7:**
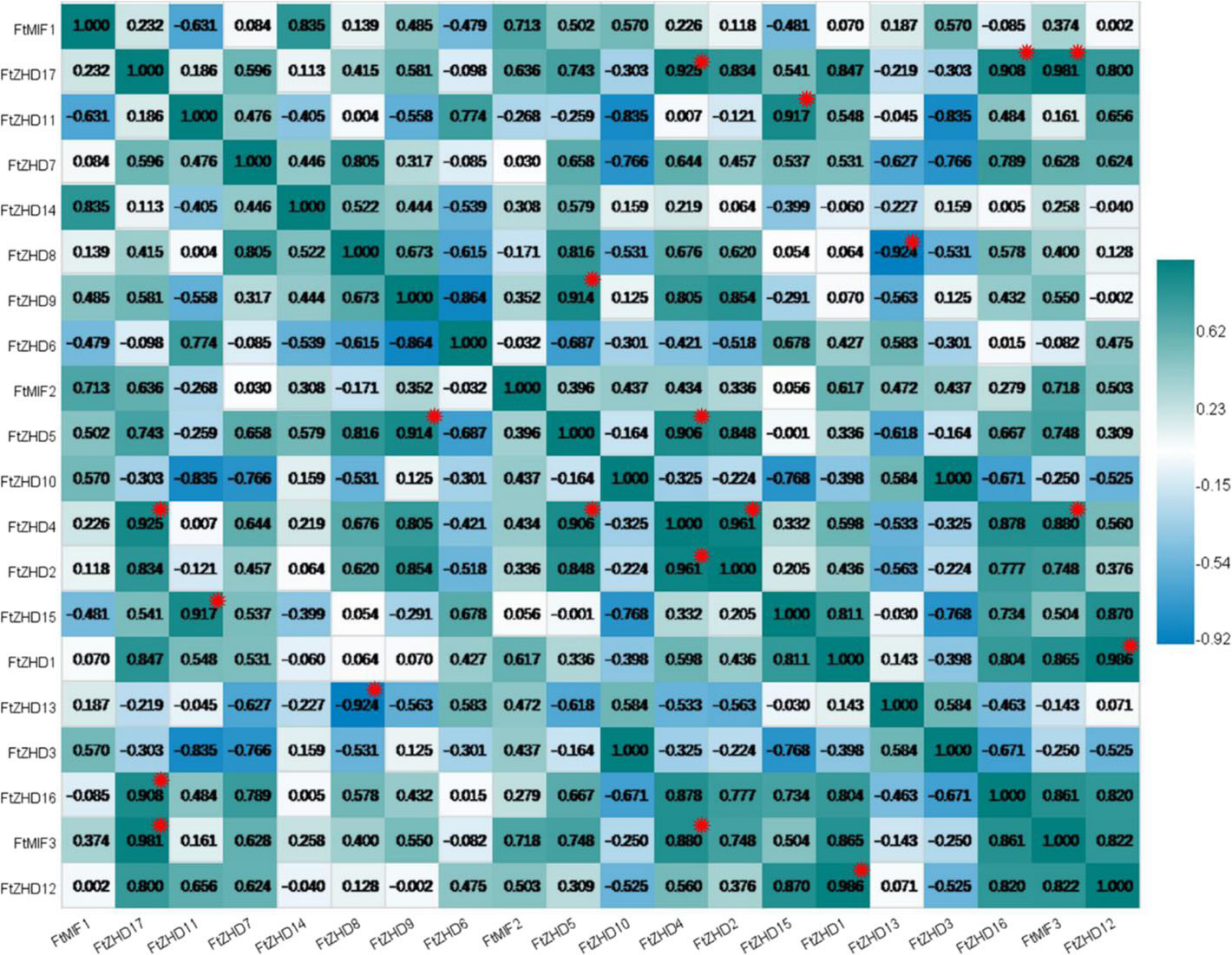
The correlation between the gene expression patterns of *FtZF-HDs*. Green: positively correlated; Blue: negatively correlated. *(red) indicates a significant correlation at the 0.05 level

We also studied the correlations among the *FtZF-HD* gene expression patterns in the roots, stems, flowers, leaves and fruits of Tartary buckwheat. Multiple pairs of genes had a strong correlation (0.880–0.986): *FtZHD 17* and *FtMIF3*, *FtZHD 17* and *FtZHD 16, FtZHD 17* and *FtZHD4, FtZHD 11* and *FtZHD* 15, *FtZHD 9* and *FtZHD 5, FtZHD 5* and *FtZHD 4, FtZHD 4* and *FtMIF3*, *FtZHD 4* and *FtZHD 2,* and *FtZHD 1* and *FtZHD 12* (Fig. 7).

### Differential expression of the *FtZF-HD* genes during fruit development of Tartary buckwheat

**Fig. 8:**
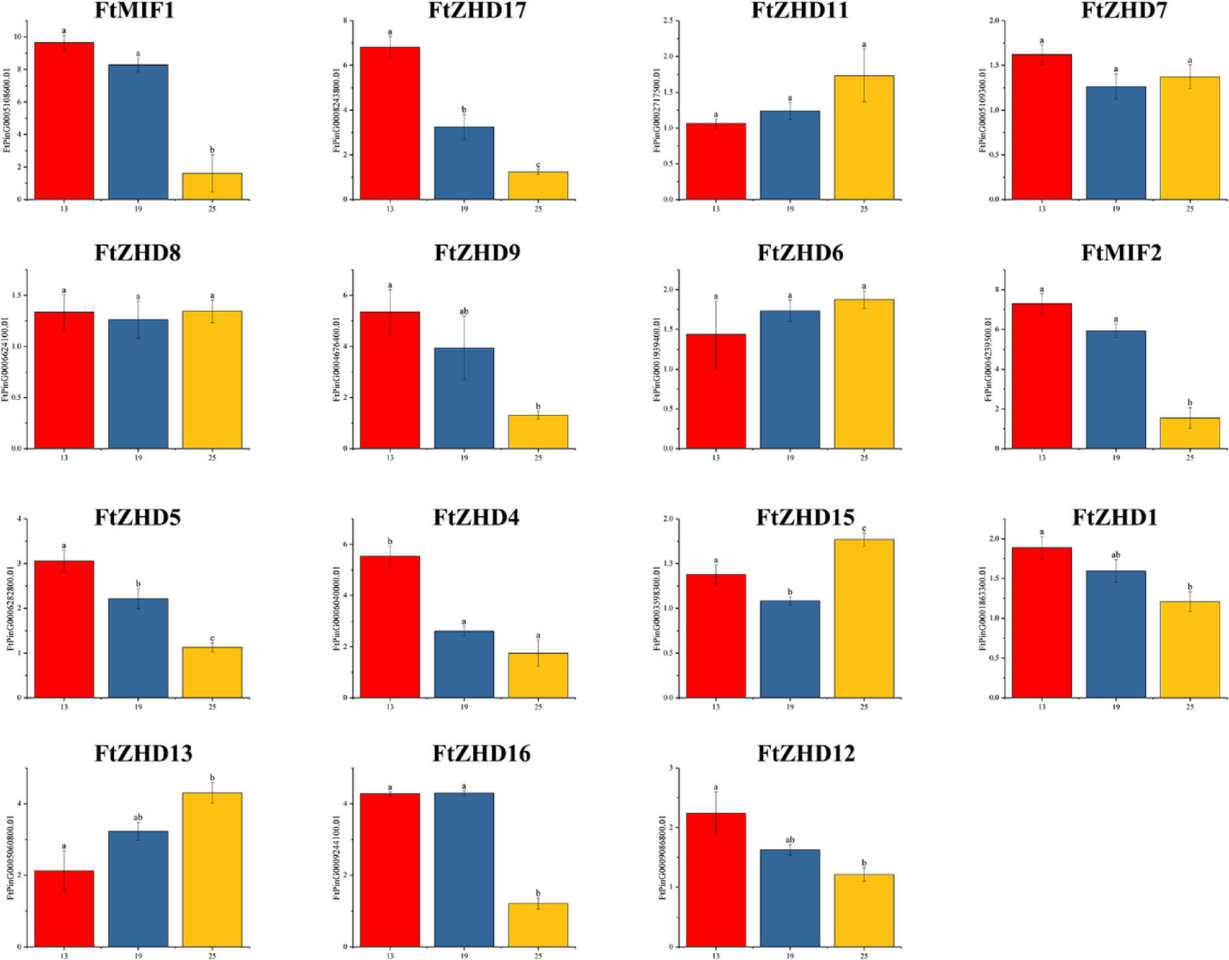
Gene expression of 15 Tartary buckwheat *ZF-HD* genes during fruit development. The expression patterns of 15 Tartary buckwheat *ZF-HD* genes in the flower, leaf, root, stem and fruit tissues were examined by a qPCR assay. Error bars were obtained from three measurements. Lowercase letter(s) above the bars indicate significant differences (α = 0.05, LSD) among the treatments

To further study the expression of the *FtZF-HD* gene in buckwheat fruit, real-time PCR was used to detect the expression of the members of the gene family in the three periods of fruit development (13 days after pollination (DAP), 19 DAP and 25 DAP) [27] and to evaluate the accumulation of *FtZF-HD* transcription products in buckwheat fruits (Fig. 8). The results showed that most of the *FtZF-HD* genes were highly expressed in the first period (13 DAP) of fruit development, implying their contribution in the early stages of fruit development. The expression of 5 *FtZF-HD* genes in the third period (25 DAP) of fruit development was higher than that in other periods, which implies their contribution to fruit ripening. The expression of all *FtZF-HD* genes in the fruit during the second period (19 DAP) was not higher than that during the other two periods, although some of the *FtZF-HD* genes were highly expressed during the second period. In this study, only one *FtZF-HD* gene (FtPinG0006624100.01) was highly expressed during all three periods of fruit development, and the level of expression was stable (Fig. 8).

**Fig. 9:**
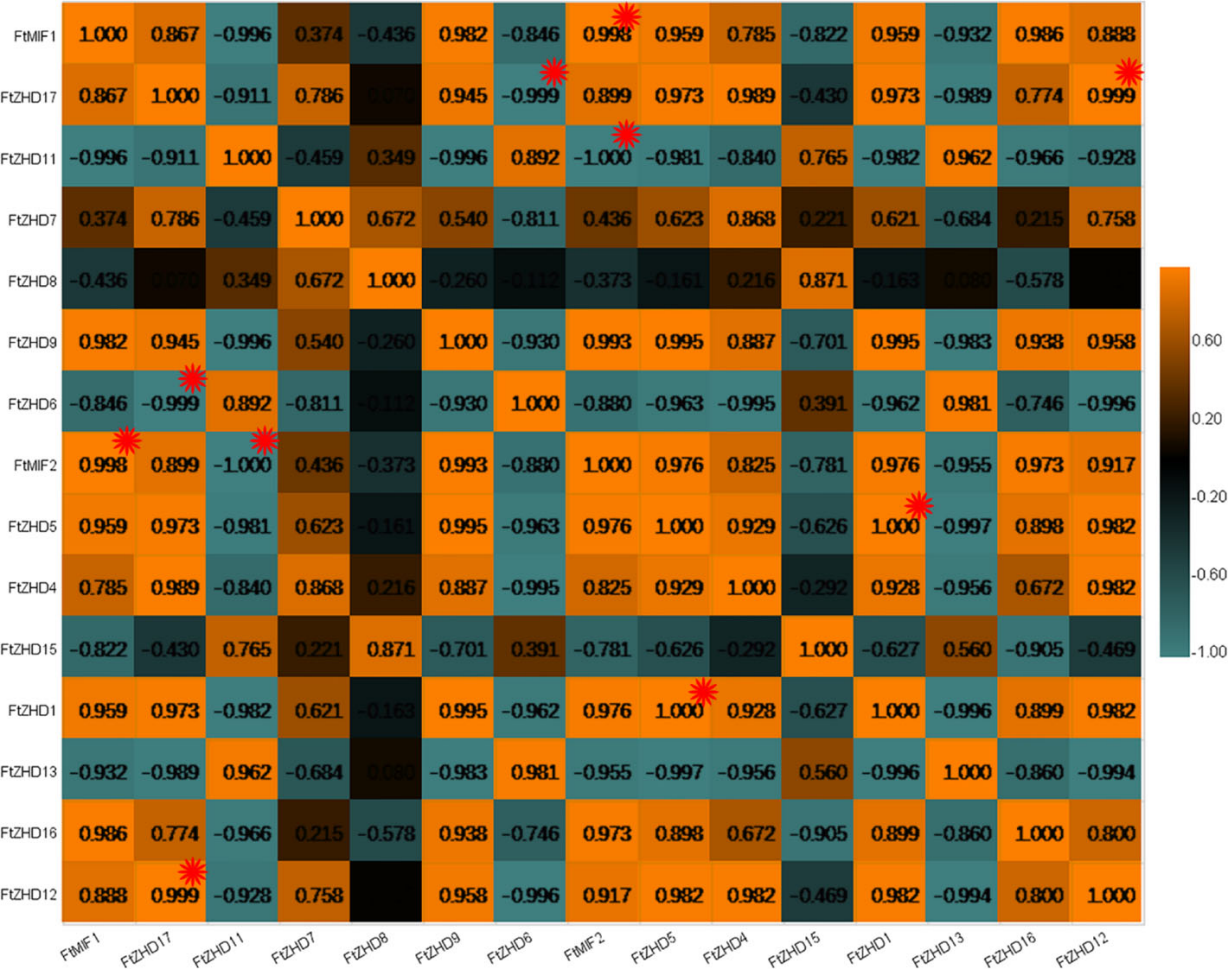
The correlation between the gene expression patterns of *FtZF-HDs* during fruit development. Orange: positively correlated; Green: negatively correlated. *(red) indicates a significant correlation at the 0.05 level

At the same time, we also studied the correlations among the expression patterns of the *FtZF-HD* genes in three stages of fruit development. Three pairs of genes had strong correlations (0.998–1.000): *FtMIF1* and *FtMIF2, FtZHD17* and *FtZHD12,* and *FtZHD12* and *FtZHD1* (Fig. 9).

## Discussion

A phylogenetic analysis and sequence analysis of the *ZHD* and *MIF* genes indicate that both genes are endemic to terrestrial plants and belong to two different groups of the same protein family (ZF-HD). Extensive research has shown that *ZHD* has been found in many terrestrial plants but not in algae [12]. We identified 20 *ZF-HD* genes in Tartary buckwheat, including 17 *ZHD* genes and 3 *MIF* genes, which was slightly higher than the number found in Arabidopsis (17). The replication of genes can amplify the number of genes. For example, four large replication events occurred in the Arabidopsis genome, and more than half of *ZF-HD* genes may be produced by genomic replication [12, 28, 29]. Gene replication mechanisms include fragment replication, tandem replication and translocation (reverse transcription and replication translocation), which is an important factor in biological evolution [30]. Among the mechanisms, fragment replication is one of the main contributors to the amplification of many gene families [31]. The analysis of the chromosome distribution of the *ZF-HD* genes in Tartary buckwheat revealed there was fragment replication, but no tandem replication, which suggests that the gene fragment replication events greatly facilitates the expansion of the *ZF-HD* gene family in plants with smaller genomes. Moreover, a phylogenetic tree with Arabidopsis also suggests an evolutionary relationship between these two species. There are three main mechanisms for the variation of the exon and intron structures of a gene (the gain or loss, exonization or pseudoexonization and insertion or deletion of exon or intron), and each mechanism leads to a gene structural difference [15, 32–34]. The presence of a few introns in the Tartary buckwheat *ZF-HD* gene may be due to the variations in the participation of these three mechanisms. To study the phylogenetic relationship between Tartary buckwheat and dicotyledon, we constructed collinear relationship maps between Tartary buckwheat and seven plant species (Fig. 4). Finally, 23 colinear *ZF-HD* gene pairs of Tartary buckwheat and soybean were identified. The number of orthologous events was far greater than that between Tartary buckwheat and rice, which was consistent with the closer evolutionary distance between Tartary buckwheat and soybean [35].

The *ZF-HD* transcription factors are involved in various biological processes such as the response of plants to abiotic stress and the development of plants by phytohormones [34, 36]. Most of the previous reports on the *ZF-HD* gene were about the regulation of the *ZF-HD* gene in response to abiotic stress, but there were few reports on plant development. Members of the *ZF-HD* gene family are expressed in floral tissues in plants (e.g. Arabidopsis) [36]. It was revealed that the *ZF-HD* gene family regulates flower development. Our research shows that the 20 *ZF-HD* genes identified from Tartary buckwheat were indeed expressed during the growth and development of plants, so there is no pseudogene. However, in the study of fruit development, we found that five genes were not involved in the regulation of fruit development. It is worth noting that *FtMIF3*, which belongs to the same subfamily as *FtMIF1* and *FtMIF2*, is not expressed in the fruit, and when the motifs of the three were compared (Fig. 2), we found that they had the same motifs. This result leads us have great interest in exploring the reasons for the differences among the genes because after comparing their protein sequences (Additional file 1: Figure S1), we found that although the three motifs are the same, the amino-acid residues at the 126th to 139th positions of the amino acid sequence encoded by the *FtMIF3* gene differ significantly from those of *FtMIF1* and *FtMIF2*. We speculate that it is highly likely that a mutation in the *FtMIF3* gene caused the protein it encodes to lose its ability to express itself in the fruit. For *FtMIF1* and *FtMIF2*, their differences in amino acid sequences have little effect, and the expression of *FtMIF1* and *FtMIF2* in fruit development has a 0.998 correlation. Although not all genes are involved in fruit development, it is equally exciting to find that 15 genes are expressed in the fruit. Before this study, the expression of the ZF-HD protein family in fruit was only reported in grape (*Vitis vinifera*) and tomato, and there was only a potential role in grape fruit development [14, 15].

We found four grape genes that were expressed in fruits in the phylogenetic trees containing multiple plants (Fig. 5) (*GSVIVT01003614001, GSVIVT01009128001, GSVIVT01012772001* and *GSVIVT 01011413001*, in groups a and b, respectively) [15]. These genes have similar to the motifs of those of Tartary buckwheat in the respective groups. For example, the genes in group a have motif1 and motif3, and the genes, except for *FtPinG006040000.01*, in group b have motif1, motif2, motif3 and motif4. It can be seen that the relationship between the grape genes and Tartary buckwheat genes in group b is closer than that in group a.

In analyzing the expression of genes in the tissues (Fig. 6), we noted that five genes are more highly expressed in reproductive organs (flowers and fruits) than in other tissues *(FtZHD1, FtZHD6, FtZHD12, FtZHD15,* and *FtZHD11*). In addition, the expression of *FtZHD1* and *FtZHD12* in the flowers was higher than that in the fruit, and the expression of *FtZHD6, FtZHD15* and *FtZHD11* in the fruit was higher than that in the flowers. Comparing their motifs (Fig. 2), we found that *FtZHD12* and *FtZHD15* have exactly the same motifs. Therefore, we speculate that there may be a functional overlap between the two genes. With the study of their expression during fruit development (Fig. 8), we found that the expression of *FtZHD12* and *FtZHD1* in the earlier stage was higher than that in the middle and late stages, the expression of *FtZHD6, FtZHD11* and *FtZHD15* in late stage was higher than that in middle stage and earlier stage, and it was discovered by a synchronous analysis of the genes (Fig. 3) that there was fragment duplication between *FtZHD6* and *FtZHD11*. Therefore, *FtZHD6* and *FtZHD11* have similar functions in the development of the reproductive organs and fruits. We also found that *FtZHD11* and *FtZHD15* have a 0.917 correlation in plant tissue development, so we speculate that *FtZHD11* and *FtZHD15* may also have functional overlaps.

The size of monocotyledonous fruit is related to endosperm development [37, 38], in dicotyledonous plants, the final size of the fruit is related to the number and size of cotyledon cells [39]. Studies have pointed out that usually the initial growth of the endosperm rather than the late growth of the embryo is mainly related to the size of the fruit [40–42]. Reports on the development of Tartary buckwheat fruit indicated that the size of Tartary buckwheat fruit is mainly related to cell division during embryonic development, and most Tartary buckwheat fruits reach the maximum state on the 13th to 25th day after pollination. [43]. Therefore, in this study, the early stage of fruit development is the key period in determining the size of the fruit. In our research on fruit development, we found that there are 8 genes that are more highly expressed in the earlier stages than in the other two stages. Fruit development is controlled by various transcriptional regulatory networks. These networks involve transcription factors, for example, members of the ARF family, which can regulate hormones such as AUX and ABA [43]. *FtARF* 2 may have the ability to integrate signals, thus prolonging the cycle of embryonic development, increasing the cycle of cell division and increasing the accumulation of storage materials in the fruit tissue [43]. The relationship between *FtMIF1, FtMIF2* and *FtZHD17* and the *FtARF* gene family needs further study; however, *FtMIF1, FtMIF2* and *FtZHD17* may be related to the fruit size of Tartary buckwheat. In addition, *FtZHD12*, which has a 0.999 correlation with *FtZHD17*, may also be related to fruit size. In conclusion, the analysis of *FtZF-HD* gene expression in the tissues and fruit laid a foundation for breeding new Tartary buckwheat varieties.

## Conclusions

20 *FtZF-HD* genes were identified in Tartary buckwheat, and the structures, evolution and expression patterns of the proteins were studied. Our findings provide a valuable basis for further analysis of the biological function of the *ZF-HD* gene family. Our study also laid a foundation for the improvement of Tartary buckwheat crops.

## Methods

### Identification of *ZF-HD* genes in Tartary buckwheat

We downloaded the Tartary buckwheat genome from the Tartary buckwheat genome project (TBGP; http://www.mbkbase.org/Pinku1/) [22]. The *ZF-HD* gene family of Tartary buckwheat was searched by two BLASTP methods. The hidden Markov model (HMM) file corresponding to the ZF-HD_dimer domain (PF04770) was downloaded from the Pfam protein family database (http://pfam.xfam.org/). The existence of ZF-HD_dimer core sequences was verified by PFAM and SMART programs. Finally, 20 genes containing *ZF-HD* domain were screened from tartary buckwheat genome. By using the tools from the ExPASy website (https://web.expasy.org/compute_pi/), we obtained the sequence length, molecular weight, isoelectric point and subcellular localization of the identified 20 *ZF-HD* proteins.

### Sequence analysis

Using the ZF-HD_dimer domain sequence of the *FtZF-HD* proteins, we used the default parameter ClustalW to compare several protein sequences. To determine the exon-intron structure of the *FtZF-HD* genes, the predicted coding sequence was compared with the corresponding full-length sequence by the Gene Structure Display Server (GSDS: http://gsds.cbi.pku.edu.cn) online program. Using the MEME online program (http:/meme.nbcr.net/meme/intro.html) to analyze the protein sequences under the following parameters: the optimum motif width was 6 ~ 200; the maximum number of motifs was 10.

### Chromosomal distribution and gene duplication of *FtZF-HD* genes

The method of *FtZF-HD* genes mapping to the chromosomes of tartary buckwheat was refer to Liu et al. [22]. Analysis of *FtZF-HD* genes replication events using Multiple collinear scanning toolkits (MCScanX). The syntenic relationship between *FtZF-HD* genes and *ZF-HD* genes from selected plants were determined by using the Dual Systeny Plotter software (https://github.com/CJ-Chen/TBtools).

### Phylogenetic analysis and classification of the FtZF-HD gene family

Using ZF-HD protein sequences (*Arabidopsis thaliana*, maize, rice and soybean) downloaded from the UniProt database (https://www.uniprot.org/) constructed phylogenetic trees. We using the NJ method of Geneious R11 to derived the phylogenetic tree. The parameters were the Jukes-Cantor model, and global alignment with free end gaps.

### Plant growth

This study used the Tartary buckwheat (XiQIAO) germplasm provided by Professor Wang Anhu of Xichang University. We planted materials in the experimental field of the College of Life Sciences of Sichuan Agricultural University (Lat. 29°97′ N, 102°97′ E, Alt. 580 m), Ya’an, Sichuan, China [22]. The samples including flower, the fruit from three (13, 19, and 25, DAP) different developmental fruit stages, and the stem, root, and leaf of mature tartary buckwheat were collected separately and quickly put into liquid nitrogen and stored at − 80 °C for further use.

### Expression analysis of the *FtZF-HD* genes using real-time PCR

Using the Primer3 software (http://bioinfo.ut.ee/primer3/) designed the RT-qPCR primers (Additional file 5: Table S4). Quantitative real-time PCR analysis was used to analyze the identified genes. Using the *FtH3* gene as an internal control, the standard RT-qPCR with SYBR Premix Ex Taq II (TaKaRa) was repeated at least three times on a CFX96 Real Time System (BioRad). The data were analyzed by the 2^−(∆∆Ct)^ method, and the relative mRNA expression data were obtained [44].

### Statistical analysis

We used Origin Pro 2018b (OriginLab Corporation., Northampton, Massachusetts, USA) statistics program to analyze all the data, and the means were compared by the least significant difference test (LSD) at significance levels of 0.05 and 0.01 [22].

## Additional files

Additional file 1: **Figure S1** Alignment of multiple FtZF-HD domain amino acid sequences. Colored amino acids indicate that multiple genes have the same amino acids at the same location. (DOCX 231 kb)

Additional file 2: **Table S1** List of the 20 *FtZF-HD* genes identified in this study. (XLS 67 kb)

Additional file 3: **Table S2** Analysis and distribution of conserved motifs in Tartary buckwheat ZF-HD proteins. (XLS 31 kb)

Additional file 4: **Table S3** One-to-one orthologous relationships between tartary buckwheat and other seven plant species. (XLS 44 kb)

Additional file 5: **Table S4** Primers of sequences. (XLS 30 kb)

## Data Availability

The genome sequences of Tartary buckwheat used for identifying the *ZF-HD* genes in this study were located in the Tartary Buckwheat Genome Project (TBGP; http://www.mbkbase.org/Pinku1/). The Tartary buckwheat accessions (XIQIAO; accessions number: CHUAN 2008013) materials used in the experiment were supplied by Professor Wang Anhu of Xichang University. The datasets supporting the conclusions of this article are included with in the article and its Supplementary files.

## Abbreviations

At: *Arabidopsis thaliana*
DAP: Days after pollination
Ft: *Fagopyrum tataricum*
Gm: *Glycine max*
HB: Homeobox
HD: Homeodomain
HD-Zip: Leucine zipper-associated HD
KNOX: Knotted-related homeobox
MIF: Mini zinc finger
PHD finger: Finger-like domain associated with an homeodomain
WOX: WUSCHEL-related homeobox
ZF-HD: Zinc finger motif-associated homeodomain
