# Supplementary material for: Genome-wide investigation of the ZF-HD gene family in Tartary buckwheat (Fagopyrum tataricum)
Source: BMC Plant Biol. 2019 Jun 11;19:248. doi: 10.1186/s12870-019-1834-7 (PMC6558689; doi:10.1186/s12870-019-1834-7)
Supplement: Supplementary file 1 — Figure S1 Alignment of multiple FtZF-HD domain amino acid sequences. Colored amino acids indicate that multiple genes have the same amino acids at the same location. (DOCX 231 kb) [file 12870_2019_1834_MOESM1_ESM.docx]

**Title:** Genome-Wide Investigation of the *ZF-HD* Gene Family in Tartary Buckwheat (*Fagopyrum tataricum*)

Moyang Liu ^†^, Xiaoxiang Wang, Wenjun Sun, Zhaotang Ma, Tianrun Zheng, Li Huang, Qi Wu, Zizhong Tang, Tongliang Bu, Chenglei Li, and Hui Chen^*^

*Sichuan Agricultural University, College of Life Science, Ya’an, China*

^†^These authors contributed equally to this work.

^*^Corresponding authors: Hui Chen, email: chenhui@sicau.edu.cn, phone (+86) 18981604486


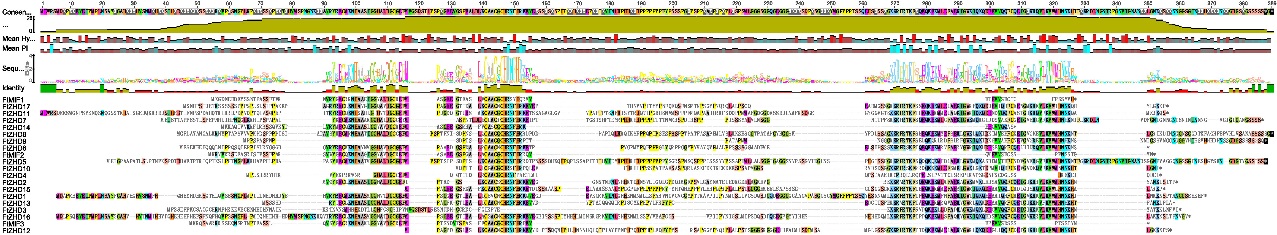


**Figure S1** Alignment of multiple FtZF-HD domain amino acid sequences. Colored amino acids indicate that multiple genes have the same amino acids at the same location.
